# Abstracts of the 52nd Workshop for Pediatric Research: Frankfurt, Germany. 27-28 October 2016

**DOI:** 10.1186/s40348-017-0071-0

**Published:** 2017-05-10

**Authors:** Rhea van den Bruck, Patrick P. Weil, Thomas Ziegenhals, Philipp Schreiner, Stefan Juranek, Daniel Gödde, Silvia Vogel, Frauke Schuster, Valerie Orth, Johannes Dörner, Daniel Pembaur, Meike Röper, Stefan Störkel, Hubert Zirngibl, Stefan Wirth, Andreas C. W. Jenke, Jan Postberg, Nikolas Boy, Jana Heringer, Gisela Haege, Esther M. Glahn, Georg F. Hoffmann, Sven F. Garbade, Peter Burgard, Stefan Kölker, Cho-Ming Chao, Faady Yahya, Alena Moiseenko, Amit Shrestha, Negah Ahmadvand, Jennifer Quantius, Jochen Wilhelm, Elie El-Agha, Klaus-Peter Zimmer, Saverio Bellusci, Christian Staufner, Stefan Kölker, Holger Prokisch, Georg F. Hoffmann, Stephan Seeliger, Matthias Müller, Andreas Hippe, Henrik Steinkraus, Roland Wauer, Burkhard Lachmann, Sigrun R. Hofmann, Christian M. Hedrich, Jakob Zierk, Farhad Arzideh, Rainer Haeckel, Wolfgang Rascher, Manfred Rauh, Markus Metzler, Sebastian Thieme, Joanna Bandoła, Cornelia Richter, Martin Ryser, Arshad Jamal, Michelle P. Ashton, Malte von Bonin, Matthias Kuhn, Christian M. Hedrich, Ezio Bonifacio, Reinhard Berner, Sebastian Brenner, Johanna Hammersen, Cristina Has, Nora Naumann-Bartsch, Daniel Stachel, Dimitra Kiritsi, Stephan Söder, Mathilde Tardieu, Markus Metzler, Leena Bruckner-Tuderman, Holm Schneider, F. Bohne, D. Langer, R. Cencic, T. Eggermann, U. Zechner, J. Pelletier, F. Zepp, T. Enklaar, D. Prawitt, Martin Pech, Markus Weckmann, Femke-Anouska Heinsen, Andre Franke, Christine Happle, Anna-Maria Dittrich, Gesine Hansen, Oliver Fuchs, Erika von Mutius, Brian G. Oliver, Matthias V. Kopp, Claudia Paret, Alexandra Russo, Johanna Theruvath, Bettina Keller, Khalifa El Malki, Nadine Lehmann, Arthur Wingerter, Marie A. Neu, Gerhold-Ay Aslihan, Wolfgang Wagner, Clemens Sommer, Torsten Pietsch, Larissa Seidmann, Jörg Faber, Felix Schreiner, Merle Ackermann, Michael Michalik, Eva Rother, Andras Bilkei-Gorzo, Ildiko Racz, Laura Bindila, Beat Lutz, Jörg Dötsch, Andreas Zimmer, Joachim Woelfle, Hendrik S. Fischer, Tim L. Ullrich, Christoph Bührer, Christoph Czernik, Gerd Schmalisch, Robert Stein, Sigrun R. Hofmann, Judith Hagenbuchner, Ursula Kiechl-Kohlendorfer, Petra Obexer, Michael J. Ausserlechner, Niki T. Loges, Adrien Tobias Frommer, Julia Wallmeier, Heymut Omran, Soner Öner-Sieben, Martina Gimpfl, Jan Rozman, Martin Irmler, Johannes Beckers, Martin Hrabe De Angelis, Adelbert Roscher, Eckhard Wolf, Regina Ensenauer, Karolina Nemes, Michael Frühwald, Martin Hasselblatt, Reiner Siebert, Uwe Kordes, Marcel Kool, Haicui Wang, Holly Hardy, Osama Refai, Katy E. S. Barwick, Holly H. Zimmerman, Joachim Weis, Emma L. Baple, Andrew H. Crosby, Sebahattin Cirak, C. Hellmuth, O. Uhl, M. Standl, J. Heinrich, E. Thiering, B. Koletzko, Lena Blümel, Kornelius Kerl, Daniel Picard, Michael C. Frühwald, Max C. Liebau, Guido Reifenberger, Arndt Borkhardt, Martin Hasselblatt, Marc Remke, D. Tews, M. Wabitsch, P. Fischer-Posovszky, Mike-Andrew Westhoff, Lisa Nonnenmacher, Julia Langhans, Lukas Schneele, Nancy Trenkler, Klaus-Michael Debatin

**Affiliations:** 1 Department of Paediatrics, HELIOS Medical Centre Wuppertal, Centre for Clinical and Translational Research (CCTR), Witten/Herdecke University Hospital, Centre for Biomedical Education and Research (ZBAF), Wuppertal, Germany; 2 Chair of Biochemistry, Theodor-Boveri-Institute at the Biocenter, University of Würzburg, Würzburg, Germany; 3 Molecular Pathology Department, HELIOS Medical Centre Wuppertal, Witten/Herdecke University Hospital, Wuppertal, Germany; 4 Department of Plastic, Reconstructive, Aesthetic and Hand Surgery, HELIOS Medical Centre Wuppertal, Witten/Herdecke University Hospital, Wuppertal, Germany; 5 Department of Surgery II, HELIOS Medical Centre Wuppertal, Witten/Herdecke University Hospital, Wuppertal, Germany; 6 Centre for Child and Adolescent Medicine, Department of General Pediatrics, Division of Neuropaediatrics and Metabolic Medicine, University Hospital Heidelberg, Im Neuenheimer Feld 430, D-69120 Heidelberg, Germany; 7 Excellence Cluster Cardio-Pulmonary System, Gießen, Germany; 8 Department for General Pediatrics and Neonatology, University Children’s Hospital, Gießen, Germany; 9 German Center for Lung Research (DZL), Gießen, Germany; 10 Department of General Pediatrics, University Children’s Hospital, University Hospital Heidelberg, 69120 Heidelberg, Germany; 11 Institute of Human Genetics, Helmholtz Zentrum München, 85764 Neuherberg, Germany; 12 Institute of Human Genetics, Technische Universität München, 81675 Munich, Germany; 13 Department of Pediatric Cardiology, Intensive Care and Neonatology, University Hospital Goettingen, 37075 Goettingen, Germany; 14 Department of Dermatology, Heinrich Heine University, 40225 Duesseldorf, Germany; 15 Department of Anesthesiology, MSP, Surgical Intensive Care Medicine, University Hospital, Charité, Campus Virchow Clinic, 13353 Berlin, Germany; 16 Department of Neonatology, Charité, University of Medicine, 10098 Berlin, Germany; 17 Clinics for children and adolescents, 86633 Neuburg/Donau, Germany; 18 Klinik und Poliklinik für Kinder- und Jugendmedizin, Universitätsklinikum Carl Gustav Carus, TU Dresden, Dresden, Germany; 19 Department of Pediatrics and Adolescent Medicine, University Hospital Erlangen, Erlangen, Germany; 20 Department of Statistics, University of Bremen, Bremen, Germany; 21 Bremer Zentrum für Laboratoriumsmedizin, Klinikum Bremen Mitte, Bremen, Germany; 22 Department of Pediatrics, University Clinic Dresden, Dresden, Germany; 23 DFG-Center for Regenerative Therapies Dresden, Cluster of Excellence, Technische Universitaet Dresden, Dresden, Germany; 24 Medical Clinic I, University Clinic Dresden, Dresden, Germany; 25 DKTK-German Cancer Consortium, Partner Site Dresden, University Clinic Dresden, Dresden, Germany; 26 DKFZ-German Cancer Research Center, Heidelberg, Germany; 27 Institute for Medical Informatics and Biometry, Faculty of Medicine, Technische Universitaet Dresden, Dresden, Germany; 28 Department of Pediatrics, University Hospital Erlangen, Erlangen, Germany; 29 Department of Dermatology, Medical Center, University of Freiburg, Freiburg, Germany; 30 Department of Pathology, University Hospital Erlangen, Erlangen, Germany; 31 Dermatologie Pédiatrique, University Hospital Grenoble, Grenoble, France; 32 Centre for Paediatrics and Adolescent Medicine, University Medical Centre, Langenbeckstr. 1, 55101 Mainz, Germany; 33 Department of Biochemistry and The Rosalind and Morris Goodman Cancer Research; Centre, McGill University, Montreal, Quebec H3G 1Y6 Canada; 34 Institute of Human Genetics, RWTH Aachen, Pauwelsstr. 30, 52074 Aachen, Germany; 35 Institute of Human Genetics, University Medical Centre, Langenbeckstr. 1, 55101 Mainz, Germany; 36 University Medical Center Schleswig-Holstein, Division Pediatric Pneumology & Allergology, Campus Lübeck, Lübeck, Germany; 37 Airway Research Center North (ARCN), Member of of the German Center of Lung Research (DZL), Borstel, Germany; 38 Institute of Clinical Molecular Biology, Christian-Albrechts-University of Kiel, Kiel, Germany; 39 Hannover Medical School, Department of Pediatric Pneumology, Allergology and Neonatology, Hannover, Germany; 40 Biomedical Research in Endstage and Obstructive Lung Disease Hannover (BREATH), Member of of the German Center of Lung Research (DZL), Hannover, Germany; 41 Ludwig-Maximilians-University Munich, Dr von Hauner Children’s Hospital, Munich, Germany; 42 Comprehensive Pneumology Center München (CPC-M), Member of of the German Center of Lung Research (DZL), Munich, Germany; 43 Woolcock Institute of Medical Research, Sydney, Australia; 44 Section of Pediatric Oncology, Children’s Hospital, University Medical Center of the Johannes Gutenberg University Mainz, Mainz, Germany; 45 Institute of Medical Biostatistics, Epidemiology and Informatics (IMBEI), University Medical Center of the Johannes Gutenberg University Mainz, Mainz, Germany; 46 Section of Pediatric Neurosurgery, Department of Neurosurgery, University Medical Center of the Johannes Gutenberg University Mainz, Mainz, Germany; 47 Devision of Neuropathology, University Medical Center of the Johannes Gutenberg University Mainz, Mainz, Germany; 48 Department of Neuropathology, University of Bonn, Bonn, Germany; 49 Institute of Pathology, University Medical Center of the Johannes Gutenberg University Mainz, Mainz, Germany; 50 UCT Mainz, Mainz, Germany; 51 Pediatric Endocrinology, Children’s Hospital, University of Bonn, Bonn, Germany; 52 Pediatric Endocrinology, Children’s Hospital, University of Cologne, Cologne, Germany; 53 Molecular Psychiatry, University Hospital Bonn, Bonn, Germany; 54 Institute for Physiological Chemistry, University Medical Center, Mainz, Germany; 55 Department of Neonatology, Charité University Medical Center, Berlin, Germany; 56 Klinik und Poliklinik für Kinder- und Jugendmedizin, Universitätsklinikum Carl Gustav Carus, TU Dresden, Dresden, Germany; 57 Department of Pediatrics II, Innsbruck, Austria; 58 Department of Pediatrics I, Medical University Innsbruck, Innsbruck, Austria; 59 Tyrolean Cancer Research Institute, Innsbruck, Austria; 60 Department of General Pediatrics, University Children’s Hospital Muenster, Muenster, Germany; 61 Experimental Pediatrics, University Children’s Hospital, Heinrich Heine University Düsseldorf, Düsseldorf, Germany; 62 Research Center, University Children’s Hospital, Ludwig-Maximilians-Universität (LMU) München, München, Germany; 63 Institute of Experimental Genetics, Helmholtz Zentrum München, München, Germany; 64 Institute of Molecular Animal Breeding and Biotechnology, Gene Center, LMU München, München, Germany; 65 Children’s Hospital Augsburg, Swabian Children’s Cancer Center, Stenglinstr. 2, 86156 Augsburg, Germany; 66 Institute of Neuropathology, University Hospital Münster, Pottkamp 2, 48149 Münster, Germany; 67 Department of Human Genetics, Institute of Human Genetics, University of Ulm, Ulm, Germany; 68 Department of Pediatric Hematology and Oncology, University Medical Center Hamburg Eppendorf, Martinistraße 52, 20246 Hamburg, Germany; 69 Division of Pediatric Neurooncology (B062), German Cancer Consortium (DKTK), German Cancer Research Center (DKFZ), Heidelberg, Germany; 70 Uniklinik Köln, Klinik für Kinderheilkunde und Jugendmedizin, Köln, Germany; 71 RILD Wellcome Wolfson Centre, Royal Devon & Exeter NHS Foundation Trust, Barrack Road, Exeter, UK; 72 University of Calgary, Calgary, Canada; 73 University of Mississippi, Medical Center of Jackson, Jackson, MS USA; 74 Uniklinik Aachen, Institut für Neuropathologie, Aachen, Germany; 75 Ludwig-Maximilian-Universität Munich, Div. Metabolic and Nutritional Medicine, Dr. von Hauner Children’s Hospital, University of Munich Medical Center, Munich, Germany; 76 Institute of Epidemiology I, Helmholtz Zentrum München- German Research Center for Environmental Health, Neuherberg, Germany; 77 Institute and Outpatient Clinic for Occupational, Social and Environmental Medicine, Inner City Clinic, University Hospital Munich, Ludwig Maximilian University of Munich, Munich, Germany; 78 Department of Pediatric Oncology, Hematology and Clinical Immunology, Heinrich Heine University Düsseldorf, Düsseldorf, Germany; 79 Division of Pediatric Neuro-Oncogenomics, German Cancer Consortium and German Cancer Research Center - partner site Essen/Düsseldorf, Düsseldorf, Germany; 80 Institute of Neuropathology, Heinrich Heine University Düsseldorf, Düsseldorf, Germany; 81 Department of Pediatric Hematology and Oncology, University Hospital Münster, Münster, Germany; 82 Swabian Childrens’ Cancer Center, Children’s Hospital Augsburg, Augsburg, Germany; 83 Department of Pediatrics and Center for Molecular Medicine, University Hospital Cologne, Cologne, Germany; 84 Institute of Neuropathology, University Hospital Münster, Münster, Germany; 85 Division of Pediatric Endocrinology and Diabetes, Department of Pediatrics and Adolescent Medicine, University Medical Center Ulm, Ulm, Germany; 86 Department of Pediatrics and Adolescent Medicine, University Medical Center Ulm, Ulm, Germany

## A1 First report of a lethal infantile autosomal recessive ITGB6V438M disorder correlating with impaired integrin α_V_β6 receptor dimerization in intestinal epithelia

### Rhea van den Bruck^1^, Patrick P Weil^1^, Thomas Ziegenhals^2^, Philipp Schreiner^2^, Stefan Juranek^2^, Daniel Gödde^3^, Silvia Vogel^3^, Frauke Schuster^4^, Valerie Orth^5^, Johannes Dörner^5^, Daniel Pembaur^1^, Meike Röper^1^, Stefan Störkel^3^, Hubert Zirngibl^5^, Stefan Wirth^1^, Andreas CW Jenke^1^, Jan Postberg^1^

#### ^1^Department of Paediatrics, HELIOS Medical Centre Wuppertal, Centre for Clinical and Translational Research (CCTR), Witten/Herdecke University Hospital, Centre for Biomedical Education and Research (ZBAF), Wuppertal, Germany; ^2^Chair of Biochemistry, Theodor-Boveri-Institute at the Biocenter, University of Würzburg, Würzburg, Germany; ^3^Molecular Pathology Department, HELIOS Medical Centre Wuppertal, Witten/Herdecke University Hospital, Wuppertal, Germany; ^4^Department of Plastic, Reconstructive, Aesthetic and Hand Surgery, HELIOS Medical Centre Wuppertal, Witten/Herdecke University Hospital, Wuppertal, Germany; ^5^Department of Surgery II, HELIOS Medical Centre Wuppertal, Witten/Herdecke University Hospital, Wuppertal, Germany

##### **Correspondence:** Jan Postberg

Rhea van den Bruck and Patrick P Weil are joint first authors

**Background**

A male dizygotic twin was delivered at a gestational age of 36 weeks in the Children’s Hospital of the Helios Medical Centre Wuppertal, Witten/Herdecke University Hospital. Contrary to his brother the boy presented dystrophic at birth (1.715 g birth weight; 150 g below 3rd percentile) and developed adverse gastrointestinal conditions within the first 2 months of life. These included chronic mucosal inflammation and oedematous lamina propria in the intestine, which contributed to intractable diarrhoea. At an age of 7 months the infant eventually died of enteral haemorrhages and liver failure. Further anamnesis revealed several similar fatalities in the familial clan with reportedly frequent parental consanguinity. Intractable chronic diarrhoea in infancy are heterogeneous disorders challenging for diagnostics and therapy. Despite extensive diagnostic approaches the etiology of many cases remains elusive. Investigating putatively underlying genetic disorders might clarify many cases.

**Results**

To contribute to the diagnosis we performed whole exome sequencing of the affected infant as well as his twin brother and parents. We identified a suspicious nonsynonymous single nucleotide polymorphism (SNP) in the integrin beta-6 gene (ITGB6G1312A) entailing a V438M substitution. This SNP is very rare in the G1000 cohort and predicted being potentially harmful. The allelic distribution in the genotyped family members fit well with an autosomal recessive inheritance scheme. We performed computational biological and molecular biological analyses on the α_V_β6 integrin receptor function suggesting that the integrin α_V_β6 dimerization could be impaired, potentially causing a loss of α_V_β6 function in wound healing and epithelial tissue integrity.

**Conclusions**

Our study provides a starting point for elucidating integrin α_V_β6 function and for understanding a pathomechanistical relevance of ITGB6V438M.

**Consent for publication**

The authors have written informed consent from the patients’ guardian/parent.

## A2 Development of neuropsychological functions in patients with glutaric aciduria type I

### Nikolas Boy^1^, Jana Heringer, Gisela Haege, Esther M. Glahn, Georg F. Hoffmann, Sven F. Garbade, Peter Burgard, Stefan Kölker

#### Centre for Child and Adolescent Medicine, Department of General Pediatrics, Division of Neuropaediatrics and Metabolic Medicine, University Hospital Heidelberg, Im Neuenheimer Feld 430, D-69120 Heidelberg, Germany

**Background**

Glutaric aciduria type I (GA-I) is an inherited metabolic disease caused by deficiency of glutaryl-CoA dehydrogenase (GCDH). Metabolic treatment according to current guideline recommendations has significantly improved neurological outcome. However, cognitive functions have not yet been studied in detail.

**Methods**

In a cross-sectional design, 30 patients detected by newborn screening (n = 13), high-risk screening (n = 3) or targeted metabolic testing (n = 14) were studied for simple reaction time (SRT), continuous performance (CP), visual working memory (VWM), visual-motor coordination (Tracking) and visual search (VS). Dystonia (n = 13 patients) was categorized using the Barry-Albright-Dystonia Scale (BADS). Patients were compared with 196 healthy controls. Developmental functions of cognitive performances were analysed using a negative exponential function model.

**Results**

BADS scores correlated with speed tests but not with tests measuring stability or higher cognitive functions without time constraints. Developmental functions of GA-I patients significantly differed from controls for SRT and VS but not for VWM and showed obvious trends for CP and Tracking. Dystonic patients were slower in SRT and CP but reached their asymptote of performance similar to asymptomatic patients and controls in all tests. Asymptomatic patients did not differ from controls, except showing significantly better results in Tracking and a trend for slower reactions in visual search. Data across all age groups of patients and controls fitted well to a model of negative exponential development.

**Conclusion**

Dystonic patients predominantly showed motor speed impairment, whereas performance improved with higher cognitive load. Patients without motor symptoms did not differ from controls. Developmental functions of cognitive performances were similar in patients and controls. Performance in tests with higher cognitive demand might be preserved in GA-I, even in patients with striatal degeneration.

This abstract has already been published [1].

**Reference**

1. Boy N, Heringer J, Haege G, Glahn EM, Hoffmann GF, Garbade SF, Kölker S, Burgard P. A cross-sectional controlled developmental study of neuropsychological functions in patients with glutaric aciduria type I. *Orphanet J Rare Dis* 2015;10:163.

## A3 *Fgf10* deficiency leads to disturbed formation of alveolar epithelial cell type II (AEC II) which causes lethality in a mouse model of bronchopulmonary dysplasia

### Cho-Ming Chao^1,2,3^, Faady Yahya^1,3^, Alena Moiseenko^1,3^, Amit Shrestha^1,3^, Negah Ahmadvand^1,3^, Jennifer Quantius^1,3^, Jochen Wilhelm^1,3^, Elie El-Agha ^1,3^, Klaus-Peter Zimmer^2^, Saverio Bellusci^1,3^

#### ^1^Excellence Cluster Cardio-Pulmonary System, Gießen, Germany; ^2^Department for General Pediatrics and Neonatology, University Children’s Hospital, Gießen, Germany; ^3^ German Center for Lung Research (DZL), Gießen, Germany

##### **Correspondence:** Cho-Ming Chao

**Background**

Inflammation-induced FGF10 protein deficiency is associated with bronchopulmonary dysplasia (BPD), a chronic lung disease of prematurely born infants characterized by arrested alveolar development. So far, experimental evidence for a direct role of FGF10 in BPD is lacking.

**Methods and Results**

Using the hyperoxia-induced neonatal lung injury as a mouse model of BPD, the impact of *Fgf10* deficiency in *Fgf10(+/−)* vs. *Fgf10(+/+)* pups was investigated. In normoxia, no lethality of *Fgf10(+/+)* and *Fgf10(+/−)* pups is observed. By contrast, 100% of *Fgf10(+/−)* pups died within 8 days of hyperoxic injury, with lethality starting at day 5, while *Fgf10(+/+)* pups were all alive. Lungs of pups from the two genotypes were collected on postnatal day 3 following normoxia or hyperoxia exposure for further analysis. In hyperoxia, *Fgf10(+/−)* lungs exhibited increased hypoalveolarization. Analysis by FACS of the *Fgf10(+/−) vs.* control lungs in normoxia, revealed decreased alveolar epithelial type II (AECII) cells over total EpCAM-positive cells ratio. In addition, gene array analysis indicated reduced AECII and increased AECI transcriptomic signatures in isolated AECII cells from *Fgf10(+/−)* lungs. Such imbalance in differentiation is also seen in hyperoxia and associated with reduced mature surfactant protein B and C expression. Attenuation of FGFR2b ligands activity postnatally in the context of hyperoxia also leads to increased lethality with decreased surfactant expression.

**Conclusions**

*Fgf10* deficiency affects quantitatively and qualitatively the formation of AECII cells. In addition, FGFR2b ligands (including FGF10) are also important for repair after hyperoxia exposure in neonates. Deficient AECII cells could be an additional complication for patients with BPD.

## A4 Unraveling the genetic cause of indeterminate pediatric acute liver failure

### Christian Staufner,^1^ Stefan Kölker,^1^ Holger Prokisch,^2,3^ Georg F. Hoffmann^1^

#### ^1^Department of General Pediatrics, University Children’s Hospital, University Hospital Heidelberg, 69120 Heidelberg, Germany; ^2^Institute of Human Genetics, Helmholtz Zentrum München, 85764 Neuherberg, Germany; ^3^ Institute of Human Genetics, Technische Universität München, 81675 Munich, Germany

**Background**

Acute liver failure (ALF) in infancy is a severe but life threatening event that remains indeterminate in about 50% despite extensive diagnostic workup. Growing evidence suggests that a prominent part of the unsolved cases are due to rare metabolic or genetic causes, some of which are still to be discovered.

**Methods**

Patients with indeterminate pediatric ALF are studied via whole exome sequencing. Functional studies are done to confirm the pathogenicity of identified variants and to investigate underlying disease mechanisms, including studies on patient fibroblasts and zebrafish. Deep clinical phenotyping is performed to characterize newly identified disease entities. Patients are recruited through individual study center cohorts and via national and international clinical studies on pediatric ALF, including an ongoing ESPED-study on ALF in infancy and adolescence.

**Results**

To date, mutations in *NBAS* and *IARS* have been identified as previously unknown genetic causes of infantile hepatopathy leading to ALF [1–3]. The corresponding disease phenotypes have been studied in 14 (*NBAS*) and 3 (*IARS*) patients. NBAS-deficiency is a relatively frequent cause of recurrent pediatric ALF and leads to a multisystemic phenotype with a broad clinical spectrum including short stature and facial dysmorphism. NBAS-related ALF is fever dependent and related to ER stress [1,2].

Apart from the hepatic phenotype, mutations in *IARS* cause a multisystemic disease with growth retardation with prenatal onset, intellectual disability, and muscular hypotonia. Zebrafish studies demonstrate an important role for *IARS* in embryogenesis [3].

**Conclusion**

Exome sequencing approaches, together with deep clinical phenotyping and functional testing in cell and animal model systems, allow unraveling the genetic cause of previously undetermined pediatric ALF, including the identification of new genetic diseases.

**References**

[1] Haack TB, Staufner C, Kopke MG, Straub BK, Kolker S, Thiel C et al. Biallelic Mutations in NBAS Cause Recurrent Acute Liver Failure with Onset in Infancy. *American Journal of Human Genetics. 2015* 97(1): 163–169.

[2] Staufner C, Haack TB, Kopke MG, Straub BK, Kolker S, Thiel C et al. Recurrent acute liver failure due to NBAS deficiency: phenotypic spectrum, disease mechanisms, and therapeutic concepts. *Journal of Inherited Metabolic Disease 2016* 39(1): 3–16.

[3] Kopajtich R, Murayama K, Janecke AR, Haack TB, Breuer M, Knisely AS et al. Biallelic IARS Mutations Cause Growth Retardation with Prenatal Onset, Intellectual Disability, Muscular Hypotonia, and Infantile Hepatopathy. *American Journal of Human Genetics* 2016 99(2): 414–422.

## A5 Nebulized surfactant as a therapy of the respiratory distress syndrome (RDS) –results of an animal experiment

### Stephan Seeliger^1,5^, Matthias Müller^1^, Andreas Hippe^2^, Henrik Steinkraus^3^, Roland Wauer^4^, Burkhard Lachmann^3^

#### ^1^Department of Pediatric Cardiology, Intensive Care and Neonatology, University Hospital Goettingen, 37075 Goettingen, Germany; ^2^Department of Dermatology, Heinrich Heine University, 40225 Duesseldorf, Germany; ^3^Department of Anesthesiology, MSP, Surgical Intensive Care Medicine, University Hospital; Charité, Campus Virchow Clinic, 13353 Berlin, Germany; ^4^Department of Neonatology, Charité, University of Medicine, 10098 Berlin, Germany; ^5^ Clinics for children and adolescents, 86633 Neuburg/Donau, Germany

In the past, attempts to establish nebulization of surfactant as a therapy of RDS couldn’t gain acceptance due to loss of surfactant activity and low intrapulmonary deposition. This study analyzes, whether modern aerosol drug delivery nebulizers (ADDN, Aeroneb solo®) lead to adequate surfactant (Alveofact®) activity *in vitro* and *in vivo* and a sufficient intrapulmonary deposition is ensured.

Suspensions containing surfactant concentrations of 12.25, 22.5 or 45 mg/ml were prepared. (1) *In vitro* experiments: Measurement of surface tension of suspensions using a Wilhelmy plate before/after nebulization. (2.1) *In vivo*: Using a RDS rat model (rats undergoing repetitive lung lavage), surfactant was applied either with an ADDN (study group) or as a bolus (control group) and its biological activity (index of oxygenation, PaO2/FlO2) was determined. In particular, surfactant suspensions were either applied as a bolus (control; total dose 37.25, 75 and 150 mg/kg body mass, over 1 min) or as a nebulized suspension using the stated suspensions (study group; 0.2-0.4 ml/min, over 30, 60, 90 and 120 min) to 6 animals per experiment.

(2.2) A fluorescent dye was added to Surfactant suspensions. Macro and microscopic comparison of intrapulmonary surfactant deposition was examined between control and study group.

There was no discernible difference between data of surface tension before and after surfactant nebulization. Index of oxygenation (PaO2/FlO2) was slightly increased using nebulization, additionally a more homogenous deposition of nebulized surfactant compared to bolus was observed. Observed parameters showed time and dose dependency of nebulization.

For the first time, we could show in these experiments that modern aerosol drug delivery nebulizers do not interfere with the biological activity of natural lyophilized surfactant, but that an increase of oxygenation and a more homogenous deposition of intrapulmonary surfactant can be achieved, if compared to established delivery techniques by bolus.

## A6 Molecular pathophysiology of chronic nonbacterial osteomyelitis

### Sigrun R. Hofmann, Christian M. Hedrich

#### Klinik und Poliklinik für Kinder- und Jugendmedizin, Universitätsklinikum Carl Gustav Carus, TU Dresden, Dresden, Germany

##### **Correspondence:** Christian M. Hedrich

**Background**

Chronic nonbacterial osteomyelitis (CNO) is an autoinflammatory bone disorder that can cause pain, structural bone damage, and inflammatory involvement of adjacent structures, including nerves and vessels. Since children and adolescents are most commonly affected, CNO can impact development. Previously, we demonstrated that monocytes from CNO patients fail to produce the immune-modulatory cytokine IL-10, which was caused by reduced activation of protein kinases ERK1 and 2. This resulted in impaired recruitment of the transcription factor Sp-1 to the *IL10* promoter and altered epigenetic remodeling. Here, we asked whether IL-10-related cytokines IL-19 and IL-20 are dysregulated in CRMO and, if so, what the specific effects may be.

**Results**

In addition to IL-10, the immune-regulatory cytokine IL-19 and the proinflammatory cytokine IL-20 are dysregulated in monocytes from CRMO patients. Forcing or knocking down gene expression and applying ChIP, we demonstrated that *IL10* and *IL19* undergo *trans*-activation by Sp-1. Using ChIP and MeDIP, we showed that IL10, IL19, and IL20 undergo epigenetic (dys-)regulation. Co-culturing primary human monocytes with cytokines, we demonstrated that reduced expression of IL-10, and IL-19 in CRMO results in increased inflammasome activity and IL-1β expression and release. Increased expression of IL-20 had no effect on inflammasome activity.

**Conclusions**

Altered expression of IL-10 and IL-19 contributes to inflammasome activation and a pro-inflammatory phenotype of monocytes in CNO. Since IL-10-deficient mice develop severe inflammatory bone-loss in arthritis models, which is mediated by inflammasome activation and IL-1 release, our observations contribute to a better understanding of the molecular pathophysiology of CNO. Cytokine dysregulation and inflammasome activation provide promising molecular targets in the search for disease biomarkers and future therapeutic interventions.

## A7 A data-mining framework for pediatric reference intervals

### Jakob Zierk^1^, Farhad Arzideh^2^, Rainer Haeckel^3^, Wolfgang Rascher^1^, Manfred Rauh^1^, Markus Metzler^1^

#### ^1^Department of Pediatrics and Adolescent Medicine, University Hospital Erlangen, Erlangen, Germany; ^2^ Department of Statistics, University of Bremen, Bremen, Germany; ^3^ Bremer Zentrum für Laboratoriumsmedizin, Klinikum Bremen Mitte, Bremen, Germany

**Background**

Pediatric laboratory test results have to be interpreted in the context of interindividual variation and age- and sex-dependent dynamics. Reference intervals as presently defined for separate age groups can only approximate the age-related dynamics encountered in pediatrics, and disregard the gradual transition between healthy and pathologic values. Interpretation of laboratory test results using continuous percentile charts from birth to adulthood allows accurate consideration of age-dependent dynamics, and enables comparison of individual test results to the distribution of values found in healthy children. However, the ethical and practical challenges when defining reference intervals using a population of healthy community children have restricted the creation of such percentile charts using conventional approaches, resulting in limitations when clinical decisions are based on laboratory test results.

**Methods**

We developed a data-mining algorithm to create percentile charts for laboratory analytes using laboratory data collected during clinical care of patients. A continuous density function is estimated and allows identification of the proportion of healthy samples in the clinical dataset containing both healthy and pathological samples. Based on this function, percentile charts from birth to adulthood are created.

**Results**

We analyzed clinical databases from six German tertiary care centers and one German laboratory service provider to create percentile charts which can be used as reference intervals for > 20 hematological and biochemical analytes.

**Conclusions**

The established framework enables the construction of continuous pediatric percentile charts for laboratory analytes by applying a data-mining approach to clinical databases. The resulting percentile charts enable a more precise consideration of pediatric dynamics in clinical decision making, and lay the foundations for more advanced test result interpretation in pediatric laboratory medicine.

## A8 The low affinity neurotrophin receptor p75NTR controls the immunological function of plasmacytoid dendritic cells

### Sebastian Thieme^1^, Joanna Bandoła^1^, Cornelia Richter^1^, Martin Ryser^1^, Arshad Jamal^1^, Michelle P. Ashton^2^, Malte von Bonin^3,4,5^, Matthias Kuhn^6^, Christian M. Hedrich^1^, Ezio Bonifacio^2^, Reinhard Berner^1^, Sebastian Brenner^1,2^

#### ^1^Department of Pediatrics, University Clinic Dresden, Dresden, Germany; ^2^DFG-Center for Regenerative Therapies Dresden, Cluster of Excellence, Technische Universitaet Dresden, Dresden, Germany; ^3^Medical Clinic I, University Clinic Dresden, Dresden, Germany; ^4^DKTK-German Cancer Consortium, Partner Site Dresden, University Clinic Dresden, Dresden, Germany; ^5^DKFZ-German Cancer Research Center, Heidelberg, Germany; ^6^Institute for Medical Informatics and Biometry, Faculty of Medicine, Technische Universitaet Dresden, Dresden, Germany

Plasmacytoid dendritic cells (pDCs) and neurotrophins play an important role in inflammatory diseases. We document, for the first time, the expression and immune-regulatory function of the low affinity neurotrophin receptor p75NTR by murine and human pDCs. Binding of neurotrophin nerve growth factor (NGF) to p75NTR results in attenuated interferon α secretion in response to Toll-like receptor-9 activation by CpG A, but increased secretion of interleukin (IL) -6 in response to CpG B. In the presence of NGF, murine pDCs stimulated the proliferation of CD4+ T cells and secretion of tumor necrosis factor-α and IL-6 in a p75NTR-dependent manner, whereas CD8+ T cells exhibited the opposite characteristics. These effects were mediated by differential phosphorylation of IRF3, IRF7, IKKα/β and c-Jun. Using a mouse model of ovalbumin-induced asthma, we showed that p75NTR expression by pDCs was essential for mediating allergic inflammation characterized by increased IL-4, IL-5, and IL-13 secretion; eosinophilia; lung tissue inflammation; and Goblet cell hyperplasia. Stimulation of pDCs with NGF exacerbated allergic symptoms. Further, NGF stimulation of human pDCs from patients with asthma aggravated allergen-specific T cell proliferation and IL-5 secretion. Further, NGF treatment of pDCs significantly aggravated Graft-versus-Host disease in a xenotransplantation model and delayed autoimmune diabetes in RIP-CD80GP mice. Our findings underline the global impact of p75NTR modulation on pDCs for immune regulation and its great therapeutic potential.

## A9 Genotype, clinical course, and therapeutic decision-making in 76 infants with severe generalized junctional epidermolysis bullosa

### Johanna Hammersen^1^, Cristina Has^2^, Nora Naumann-Bartsch^1^, Daniel Stachel^1^, Dimitra Kiritsi^2^, Stephan Söder^3^, Mathilde Tardieu^4^, Markus Metzler^1^, Leena Bruckner-Tuderman^2^, Holm Schneider^1^

#### ^1^Department of Pediatrics, University Hospital Erlangen, Erlangen, Germany; ^2^Department of Dermatology, Medical Center, University of Freiburg, Freiburg, Germany; ^3^Department of Pathology, University Hospital Erlangen, Erlangen, Germany; ^4^Dermatologie Pédiatrique, University Hospital Grenoble, Grenoble, France

**Aim**

Severe generalized junctional epidermolysis bullosa (JEB), a lethal hereditary blistering disorder, is usually treated by palliative care. Allogeneic stem cell transplantation (SCT) has been proposed as a therapeutic approach, yet without clinical evidence. The aim of this study was to gain further insight into the natural history of severe generalized JEB and to evaluate therapeutic decision-making and outcome of SCT in a large cohort of patients.

**Methods**

Decision-making was evaluated retrospectively in 76 patients with severe generalized JEB born in the years 2000–2015. The diagnosis was based on the absence of laminin-332 in skin biopsies. Two patients were treated with haploidentical stem cells from bone marrow or peripheral blood. Skin samples were investigated by immunofluorescence analyses.

**Results**

With an incidence of 1/150,000, severe generalized JEB occurred more often than published previously. Eleven as yet unreported mutations in the laminin-332 genes were detected. Although patients homozygous for the *LAMB3* mutation c.1903C > T lived longer than the others, life expectancy was greatly diminished (10.8 vs. 4.6 months). Most patients failed to thrive. In two patients with initially normal weight gain, the decision for SCT was made. Despite transiently increasing skin erosions, the clinical status of both subjects stabilized for several weeks after SCT, but finally deteriorated. Graft cells, but no laminin-332 were detected in skin biopsies. The patients died 96 and 129 days after SCT, respectively, one of them after receiving additional skin grafts.

**Conclusion**

Treatment of severe generalized JEB by SCT is a last-ditch attempt still lacking proof of efficacy.

This abstract has already been published [1] and is reproduced here with kind permission of Elsevier.

**Reference**

1. Hammersen J, Has C, Naumann-Bartsch N, Stachel D, Kiritsi D, Söder S, Tardieu M, Metzler M, Bruckner-Tuderman L, Schneider H. Genotype, Clinical Course, and Therapeutic Decision Making in 76 Infants with Severe Generalized Junctional Epidermolysis Bullosa. J Invest Dermatol 2016. 136 (11): 2150–2157. doi: 10.1016/j.jid.2016.06.609.

## A10 New mechanistic insight into the methylation maintenance at the H19-ICR1 with implications on imprinting diseases and different tumour entities

### Bohne F.^1^, Langer D.^1^, Cencic R.^2^, Eggermann T.^3^, Zechner U.^4^, Pelletier J.^2^, Zepp F.^1^, Enklaar T.^1^, Prawitt D.^1^

#### ^1^ Centre for Paediatrics and Adolescent Medicine, University Medical Centre, Langenbeckstr. 1, 55101 Mainz, Germany; ^2^ Department of Biochemistry and The Rosalind and Morris Goodman Cancer Research; Centre, McGill University, Montreal, Quebec, H3G 1Y6, Canada; ^3^ Institute of Human Genetics, RWTH Aachen, Pauwelsstr. 30, 52074, Aachen, Germany; ^4^ Institute of Human Genetics, University Medical Centre, Langenbeckstr. 1, 55101 Mainz, Germany

The correct dosage of imprinted genes is important for normal development and disturbances of genomic imprinting often manifest as developmental disorders or are causative for cancer. To ensure the correct dosage of imprinted genes, the selective maintenance of genomic methylation imprints in allele-specifically methylated regions, so-called Imprinting Control Regions (ICRs), is required for the uniparental expression. To date only the zinc finger protein ZFP57 has been shown to be a factor necessary for maintaining the DNA methylation memory at multiple ICRs during early development, except for the H19/IGF2-ICR (ICR1). However, genomic imprinting in the ICR1 and the consequent transcriptional regulation of H19 and IGF2 is frequently disturbed in the Beckwith-Wiedemann Syndrome, Silver-Russell Syndrome and a range of pediatric tumors, demanding a mechanistic understanding of epigenetic control for this region. We identified Kaiso (ZBTB33) as an indispensable factor for methylation maintenance in human ICR1 in primary human fibroblasts. Kaiso is known to bind unmethylated DNA motifs as well as methylated CGCG sequences. An unmethylated recognition motif is solely present in the B4 repeat of the ICR1. By using EMSA and ChIP techniques we show that Kaiso binds to the human ICR1. We utilized primary human fibroblasts to probe for the functional relevance of Kaiso-binding to the ICR1 by using two complementary approaches: a lentivirally transduced stable knock-down of Kaiso and a selective Kaiso binding inhibition by a CRISPR/Cas9 mediated genomic editing of the solitary binding site in the ICR1. Results of both strategies reveal that the inhibition of Kaiso binding to the ICR1 has a profound impact on ICR1 DNA methylation maintenance and the coordinated expression of H19 and IGF2, but not on the methylation of the neighboring cluster of imprinted genes CDKN1C and KvLQT1OT1.

## A11 RV-infection modifies DNA-methylation in nasal airway epithelium cells in asthmatics and non-asthmatics children

### Martin Pech^1,2^, Markus Weckmann^1,2^, Femke-Anouska Heinsen^3^, Andre Franke^3^, Christine Happle^4,5^, Anna-Maria Dittrich^4,5^, Gesine Hansen^4,5^, Oliver Fuchs^6,7^, Erika v. Mutius^6,7^, Brian G. Oliver^8^, Matthias V. Kopp^1,2^

#### ^1^University Medical Center Schleswig-Holstein, Division Pediatric Pneumology & Allergology, Campus Lübeck, Lübeck, Germany; ^2^Airway Research Center North (ARCN), Member of of the German Center of Lung Research (DZL), Borstel, Germany; ^3^Institute of Clinical Molecular Biology, Christian-Albrechts-University of Kiel, Kiel, Germany; ^4^Hannover Medical School, Department of Pediatric Pneumology, Allergology and Neonatology Hannover, Germany; ^5^Biomedical Research in Endstage and Obstructive Lung Disease Hannover (BREATH), Member of of the German Center of Lung Research (DZL), Hannover, Germany; ^6^Ludwig-Maximilians-University Munich, Dr von Hauner Children’s Hospital, Munich, Germany; ^7^Comprehensive Pneumology Center München (CPC-M), Member of of the German Center of Lung Research (DZL), Munich, Germany; ^8^Woolcock Institute of Medical Research Sydney, Australia

##### **Correspondence:** Martin Pech

In children, asthma is one of the most common chronic diseases with increasing prevalence. Human rhinovirus infection (HRVI) plays an important role in asthma exacerbations and is thought to be involved in the asthma development during childhood. McErlean et al. reported that HRVI changes the epigenetic gene regulatory mechanism of DNA methylation in nasal epithelium cells. We hypothesized that HRVI in the epithelium initiate a different methylation pattern in asthmatic and non-asthmatic children.

The analyzed nasal epithelium cells were collected by a nasal brushing of asthmatic (AST) and non- asthmatic (non-AST) children during a KIRA (German: *Kinder-Register Asthma,* Ethical approval was obtained from the Ethical Committee of the University of Luebeck) cohort visit. The cells were cultured up to passage 2 in BEGM (Lonza) and infected for 48 h with RV-16 using MOI 10 (multiplicities of infection). The genome wide DNA-methylation was analyzed using the HumanMethylation450 BeadChip Kit (Illumina). Flow (Partek), Partek Genomics Suite (Partek) and Prism (graphPad) were used for statistical data analysis, p-values include no false positive correction.

The overlap of viral modified DNA methylation sites in AST and non-AST is with 211 CpGs (p < 0.05) very limited. In the AST we detected 6922 (p < 0.05) viral altered CpGs which were not modified after HRVI in non-AST. Furthermore 6569 CpGs (p < 0.05) showed a viral induced DNA methylation modification in non-AST but not in AST subjects. Vanin-1 showed a decreased DNA methylation after HRVI specifically in AST (p =0.012).

The *in-vitro* results show a specific effect of HRVI on the genome wide DNA methylation in nasal epithelium. We confirmed the decreased DNA methylation of Vanin-1 as reported by Xiao et al., involved in corticosteroid therapy success. This data suggests that HRVI induced methylation may influence the asthma pathogenesis respectively the treatment.

## A12 Activation of the Basal Cell Carcinoma pathway in a patient with CNS HGNET-BCOR diagnosis: consequences for personalized targeted therapy

### Claudia Paret^1^, Alexandra Russo^1^, Johanna Theruvath^1^, Bettina Keller^1^, Khalifa El Malki^1^, Nadine Lehmann^1^, Arthur Wingerter^1^, Marie A. Neu^1^, Gerhold-Ay Aslihan^2^, Wolfgang Wagner^3^, Clemens Sommer^4^, Torsten Pietsch^5^, Larissa Seidmann^6^, Jörg Faber^1,7^

#### ^1^Section of Pediatric Oncology, Children’s Hospital, University Medical Center of the Johannes Gutenberg University Mainz, Mainz, Germany; ^2^Institute of Medical Biostatistics, Epidemiology and Informatics (IMBEI), University Medical Center of the Johannes Gutenberg University Mainz, Mainz, Germany; ^3^Section of Pediatric Neurosurgery, Department of Neurosurgery, University Medical Center of the Johannes Gutenberg University Mainz, Mainz, Germany; ^4^Devision of Neuropathology, University Medical Center of the Johannes Gutenberg University Mainz, Mainz, Germany; ^5^Department of Neuropathology, University of Bonn, Bonn, Germany; ^6^Institute of Pathology, University Medical Center of the Johannes Gutenberg University Mainz, Mainz, Germany; ^7^UCT Mainz, Mainz, Germany

Claudia Paret and Alexandra Russo are contributed equally to the work

**Background**

High-grade neuroepithelial tumor of the central nervous system with BCOR alteration (CNS HGNET-BCOR) is a recently described new tumor entity with dismal prognosis and no standard therapies exist so far.

**Aims**

The objective of this study was to identify and validate pathways deregulated in CNS HGNET-BCOR as a basis for targeted therapy approaches.

**Methods**

We applied transcriptome sequencing and Ingenuity Pathway Analysis to identify pathways deregulated in a patient with CNS HGNET-BCOR diagnosis. We isolated and characterized a cell line from a inoculation metastasis of the patient. The cell line was used to test FDA-approved drugs predicted to interfere with the deregulated pathways. The identified drug was included in the treatment protocol of the patient and its concentration was monitored in the liquor.

**Results**

We identified and validated the upregulation of the Sonic hedgehog and of the WNT signaling pathways in the tumor and the metastases of the patient. The activation of the pathways was conserved in the cell line isolated from the metastasis. Particularly, we detected high expression of SMO and GLI1, which are targetable with Itraconazol and Arsenic trioxide (ATO) respectively. A major effect on the viability of the patient-derived cells was obtained with ATO. Consequently, we included ATO, parallel to irradiation, in the treatment protocol. At the end of the treatment, the concentration of the arsenic in the liquor was 9.1 μg/L. The patient is currently in complete remission.

**Conclusions**

The molecular characterization of patient-derived tumors and the validation of relevant drugs in patient-derived cell culture hold the promise to identify relevant drugs for personalized targeted therapy. Our study lays the ground for the implementation of ATO in the treatment protocol of patients with CNS HGNET-BCOR.

## A13 Developmental programming of somatic growth, behavior and endocannabinoid metabolism by variation of early postnatal nutrition in a cross-fostering mouse model

### Felix Schreiner^1^, Merle Ackermann^1^, Michael Michalik^1^, Eva Rother^2^, Andras Bilkei-Gorzo^3^, Ildiko Racz^3^, Laura Bindila^4^, Beat Lutz^4^, Jörg Dötsch^2^, Andreas Zimmer^3^, Joachim Woelfle^1^

#### ^1^Pediatric Endocrinology, Children’s Hospital, University of Bonn, Bonn, Germany; ^2^Pediatric Endocrinology, Children’s Hospital, University of Cologne, Cologne, Germany; ^3^Molecular Psychiatry, University Hospital Bonn, Bonn, Germany; ^4^Institute for Physiological Chemistry, University Medical Center, Mainz, Germany

Nutrient deprivation during early development has been associated with the predisposition to metabolic disorders. Considering its interaction with metabolism, appetite and behavior, the endocannabinoid system (ECS) may represent a promising target of developmental programming.

By cross-fostering and variation of litter size, postnatal nutrition of CB6F1 mice was controlled during the lactation period (3, 6, or 10 pups/mother). After weaning and redistribution (age 21d), all pups received standard chow ad libitum. mRNA expression analyses (fat, liver, hypothalamus) were performed at age 50d, EC-concentrations in visceral fat were determined at ages 50d and 100d. Locomotor activity and behavior were analyzed by means of computer-assisted videotracking.

Body growth was permanently altered, with differences for length, weight, BMI and fat mass persisting beyond age 100d (all 3 > 6 > 10, p < 0.01). This was paralleled by hepatic IGF-I expression (p < 0.01). Distinct mRNA expression patterns for ECS key enzymes were observed in fat (EC-synthesis: 3 > 6 > 10 (DAGLα p < 0.05; NAPE-PLD p = 0.05)) and liver (EC-degradation: 3 > 6 > 10 (FAAH p < 0.05; MGL p < 0.01)). Concentrations of endocannabinoids AEA and 2-AG in fat tissue were largely comparable, except for a trend towards higher AEA in formerly overfed animals at age 100d (3 > 6 > 10, p = 0.08). In the arcuate nucleus, formerly overfed mice tended to express less EC-receptor transcripts (CB1R p < 0.05; CB2R p = 0.08) than their underfed fellows. Open-field social behavior testing revealed clear group differences, with formerly underfed mice turning out to be the most sociable animals (p < 0.01). Locomotor activity did not differ.

Our data indicate a developmental plasticity of growth, behavior and parameters of the ECS, with long-lasting impact of early postnatal nutrition. Developmental programming of the ECS in metabolically active tissues may play a role in the formation of the adult cardiometabolic risk profile following perinatal malnutrition in humans.

## A14 The influence of nasal high frequency oscillation ventilation amplitude and frequency on oropharyngeal gas conditions in a neonatal bench model

### Hendrik S. Fischer, Tim L. Ullrich, Christoph Bührer, Christoph Czernik, Gerd Schmalisch

#### Department of Neonatology, Charité University Medical Center, Berlin, Germany

**Background**

Nasal high frequency oscillation ventilation (nHFOV) is a promising mode of non-invasive respiratory support that may facilitate CO_2_ exhalation in very preterm infants. However, a recent survey among neonatologists reported highly viscous secretions and consecutive upper airway obstruction as specific side effects of nHFOV [1].

**Aims**

To investigate the influence of nHFOV frequency and amplitude on oropharyngeal temperature and humidity.

**Methods**

A bench model of neonatal nHFOV was used to simulate oropharyngeal gas conditions during spontaneous breathing through an open mouth. Oropharyngeal temperature (T), relative humidity (RH) and absolute humidity (AH) were measured using a digital thermo-hygro sensor during different nHFOV frequencies (7, 10, 13 Hz) and amplitudes (10, 20, 30 cm H_2_O), and also during nasal continuous positive airway pressure (nCPAP). Ten measurements were performed under each condition.

**Results**

All measurements were highly reproducible with intraclass correlations >0.94. The air in the model oropharynx was fully saturated (RH always >99%), but increasing nHFOV amplitudes and decreasing frequencies impacted negatively on T (p <0.001) and AH (p <0.001). The relationships were nonlinear, and there were significant interactions between the effects of frequency and amplitude on T and AH (p = 0.03, respectively). Mean T and AH were highest during nCPAP (T 34.8 ± 0.6 °C, AH 39.3 ± 1.3 g∙m^−3^) and lowest during nHFOV with a frequency of 7Hz and an amplitude of 30 cm H_2_O (T 32.4 ± 0.3 °C, AH 34.7 ± 0.5 g∙m^−3^).

**Conclusion**

Intense nHFOV settings with low frequencies and high amplitudes may put infants at an increased risk of side effects due to upper airway desiccation. Future studies need to identify other factors that impair oropharyngeal gas conditions during nHFOV, and develop strategies to optimize heated humidification.

**References**

1. Fischer HS et al. (2015) European Journal of Pediatrics 174:465–471

2. Roberts CT et al. (2016) Archives of Disease in Childhood - Fetal and Neonatal Edition 101:F248–F252.

## A15 Pyroptosome spreading during cell division as a new concept of innate immune response amplification

### Robert Stein, Sigrun R. Hofmann

#### Klinik und Poliklinik für Kinder- und Jugendmedizin, Universitätsklinikum Carl Gustav Carus, TU Dresden, Germany

##### **Correspondence:** Sigrun R. Hofmann

**Background**

A subset of patients with autoinflammatory diseases, characterized by recurrent febrile episodes and systemic inflammation of yet unknown origin harbour mutations in the *CASP1* gene, resulting in reduced enzymatic activity of caspase-1 and impaired IL-1β secretion. Thus, systemic inflammation in these individuals most likely results from the induction of alternative proinflammatory pathways, which was supported by elevated concentrations of the proinflammatory cytokine TNFα in some of these patients. Previously, we demonstrated that *CASP1* variants maintain prolonged interactions with receptor-interacting protein kinase 2 resulting in enhanced activation of NF-κB.

**Objectives**

Here we aimed to investigate whether an enzymatically inactive variant caspase-1 (p.C284A) leads to the alteration of speck formation and pyroptosis, a caspase-1 dependent proinflammatory cell death.

**Results**

Using an in vitro model of transduced immortalized murine macrophages we demonstrate that caspase-1 and ASC (apoptosis-associated speck-like protein containing a CARD) formed cytosolic macromolecular complexes (so-called pyroptosomes) that were significantly increased in number and size in cells carrying the variant p.C284A caspase-1 as compared to the WT. Using FLIM-FRET and co-immunoprecipitation we additionally show extended and more intense interaction of the variant p.C284A with ASC compared with WT caspase-1. Applying live cell imaging, we for the first time show that pyroptosomes of enzymatically inactive variant caspase-1 spread during cell division.

**Conclusions**

Proinflammatory signal transduction through propagation of specks during cell division may be a highly efficient and previously not appreciated mechanism during (auto)inflammation. Additionally, macrophages expressing variant p.C284A caspase-1 resist cell death and therefore may cause an initially weaker but altogether more persistent inflammatory stimulus.

## A16 Targeting BIRC5/Survivin by glycolysis inhibition as a novel therapeutic option in high-stage neuroblastoma

### Judith Hagenbuchner^1^, Ursula Kiechl-Kohlendorfer^1^, Petra Obexer^1,3^, Michael J. Ausserlechner^2^

#### ^1^Department of Pediatrics II, Innsbruck, Austria; ^2^Department of Pediatrics I, Medical University Innsbruck, Innsbruck, Austria; ^3^Tyrolean Cancer Research Institute, Innsbruck, Austria

The gain of chromosome 17q, a frequently found amplification in high-stage neuroblastoma, leads to overexpression of the anti-apoptotic protein BIRC5/Survivin and correlates with poor prognosis of this pediatric disease. We have shown before that Survivin-mediated resistance against chemotherapeutic agents is caused by the shutdown of mitochondrial complex I activity and a metabolic shift of neuroblastoma cells from oxidative phosphorylation to aerobic glycolysis. This increased glucose consumption sensitized tumor cells to glycolysis-inhibitors. Interestingly, in Survivin-overexpressing cells treatment with the glucose analogon 2-Deoxy-D-glucose (2DG) induces re-fusion of mitochondrial networks, which coincides with reduction on Survivin levels. We demonstrated that 2DG selectively acts on Survivin-expressing neuroblastoma cells and induces autophagic degradation of Survivin *via* activation of the E3-ubiquitin ligase Parkin, a downstream target of PINK1. Survivin degradation further releases bound Beclin-1, which enhances autophagy and cell death induction. Knock-down of Parkin or Beclin-1, however, reduces the sensitivity of Survivin-expressing neuroblastoma cells to glycolysis-inhibition. This selectivity of 2DG on Survivin offered an interesting therapeutic option, which was also confirmed in a xenograft mouse model. Together our data suggest that targeting glycolysis-metabolism in neuroblastoma with 17q amplification might be a useful, non-genotoxic strategy to overcome chemotherapy resistance in patients with high-stage disease.

This abstract has already been published [1].

**Reference**

1. Hagenbuchner J, Kiechl-Kohlendorfer U, Obexer P and Ausserlechner MJ. BIRC5/Survivin as a target for glycolysis inhibition in high-stage neuroblastoma. Oncogene. 2016 21;35(16):2052–61. doi: 10.1038/onc.2015.264.

## A17 Immunofluorescence analyses improve diagnostics of primary ciliary dyskinesia with radial spoke defects

### Niki T. Loges, Adrien Tobias Frommer, Julia Wallmeier, Heymut Omran

#### Department of General Pediatrics, University Children’s Hospital Muenster, Muenster, Germany

Primary ciliary dyskinesia (PCD) is a genetically heterogeneous recessive disorder caused by several distinct defects in genes responsible for cilia beating leading to defective mucociliary clearance often associated with randomization of left/right body asymmetry. PCD individuals with defective radial spoke heads are difficult to diagnose due to lack of gross ultrastructural defects and absence of *situs inversus*. So far only individuals with loss-of function mutations in *RSPH* genes were reported. We studied the consequences of different *RSPH9*, *RSPH4A*, and *RSPH1* mutations on the assembly of the radial spoke complex to improve diagnostics in PCD. We report 21 PCD individuals with bi-allelic mutations in *RSPH9, RSPH4A* and *RSPH1* including 7 novel mutations comprising also missense mutations as well as one amino acid deletion, and performed high-resolution immunofluorescence analysis of human respiratory cilia*.* Absence of RSPH4A due to mutations in *RSPH4A* results in deficient axonemal assembly of the radial spoke head components RSPH1 and RSPH9. *RSPH1* mutant cilia, lacking RSPH1 fail to assemble RSPH9, while *RSPH9* mutations result in axonemal absence of RSPH9 but do not affect the assembly of the other head proteins RSPH1 and RSPH4A. Interestingly, our results were identical in individuals carrying loss-of-function as well as missense mutations or one amino acid deletion. We conclude that RSPH4A is the core protein of the radial spoke head. Furthermore, antibodies targeting RSPH proteins can be utilized to diagnose PCD with RSPH defects and can help to assess pathogenicity of detected DNA variants.

## A18 Prenatal adipogenic exposure induces sex-specific programming of adipocyte development in mouse offspring

### Soner Öner-Sieben^1^, Martina Gimpfl^2^, Jan Rozman^3^, Martin Irmler^3^, Johannes Beckers^3^, Martin Hrabe De Angelis^3^, Adelbert Roscher^2^, Eckhard Wolf^4^, Regina Ensenauer^1,2^

#### ^1^Experimental Pediatrics, University Children’s Hospital, Heinrich Heine University Düsseldorf, Düsseldorf, Germany; ^2^Research Center, University Children’s Hospital, Ludwig-Maximilians-Universität (LMU) München, München, Germany; ^3^Institute of Experimental Genetics, Helmholtz Zentrum München, München, Germany; ^4^Institute of Molecular Animal Breeding and Biotechnology, Gene Center, LMU München, München, Germany

Adipogenic exposure *in utero* increases the offspring’s risk for developing overweight and metabolic diseases later in life. The aim was to study the mechanistic implications of “adipogenic programming” on adipocyte development in offspring. We used our NMRI pregnancy mouse model to induce periconceptional and gestational obesity and followed offspring outcomes in the absence of any postnatal adipogenic dietary influences including lactation. Between 6 and 16 weeks of age, offspring prenatally exposed to a maternal control diet (mat-CD) showed a physiological increase in adipocyte size in the intraabdominal adipose tissue. However, adipocytes of offspring prenatally exposed to a maternal high-calorie diet (mat-HCD) were, at age 6 weeks, more than twice as large compared to controls. Between age 6 and 16 weeks, the adipocyte size of mat-HCD males stagnated in growth, while that of mat-HCD females decreased nearly two-fold. We hypothesized that size regulation may occur during early adipose tissue development. Microarray data from 6-week-old mat-HCD female adipocytes showed enrichment of differentially expressed genes involved in downregulation of lipid metabolic processes, and transcript quantification identified a dynamic process in adipocyte regulation including downregulation of markers of adipose tissue expandability (*Cidea, Agpat2*) at 6 weeks and adipogenesis (*Scd2*) at 16 weeks of age. At adult age of 5 and 9 months, key markers of *de novo* lipogenesis were upregulated in the persistently small mat-HCD female adipocytes compared to controls, both on the transcript and enzyme activity level. Upon postnatal HCD feeding, adult females, more than males developed excess fat accumulation when prenatally exposed to a maternal adipogenic vs. control diet. Our results provide evidence of prenatal sex-specific programming of adverse adipocyte development and dysregulation of adipocyte expandability that persist into adulthood and predispose to obesity development.

## A19 The integration of molecular and clinical data defines novel risk groups in atypical teratoid/rhabdoid tumors (AT/RT)

### Karolina Nemes^1^, Michael Frühwald^1^, Martin Hasselblatt^2^, Reiner Siebert^3^, Uwe Kordes^4^, Marcel Kool^5^

#### ^1^Children’s Hospital Augsburg, Swabian Children’s Cancer Center, Stenglinstr. 2, 86156 Augsburg, Germany; ^2^Institute of Neuropathology, University Hospital Münster, Pottkamp 2, 48149 Münster, Germany; ^3^Department of Human Genetics, Institute of Human Genetics, University of Ulm, Ulm, Germany; ^4^Department of Pediatric Hematology and Oncology, University Medical Center Hamburg Eppendorf, Martinistraße 52, 20246 Hamburg, Germany; ^5^Division of Pediatric Neurooncology (B062), German Cancer Consortium (DKTK), German Cancer Research Center (DKFZ), Heidelberg, Germany

The EU-RHAB registry prospectively collects data on uniformly treated patients, to define a standard of care and to lay the foundation for phase I/II trials. 118 patients with AT/RT were evaluable. Reference evaluation of neuropathology, imaging, molecular diagnostics and treatment followed the EU-RHAB guidelines. Genetic analyses of the tumor suppressor gene *SMARCB1* (FISH, MLPA, sequencing) and Illumina 450 k methylation profiling segregated molecular subgroups.

54% of tumours were located infratentorially, 1.7% spinal only. Eight patients had synchronous tumours. In 20 of 92 patients a germ-line mutation (GLM) was detected. Metastases were observed in 44, a complete resection (GTR) was achieved in 39 patients. 71% completed EU-RHAB chemotherapy (in 30 high dose chemotherapy was applied). 80 patients received radiotherapy (RT), 18 of them with protons, and 27 patients obtained maintenance therapy. A CR was achieved in 62 patients, in 15 by surgery alone and in 47 by additional chemotherapy. Complete genetic analysis of *SMARCB1* was obtained in 68, 450 k methylation profiles in 47 patients. 5-year OS and EFS rates were significantly associated with age at diagnosis, metastasis, GLM, GTR, RT, CR, genetic subgroups of *SMARCB1* and 450 k methylation profile. Patients with mutation (frame shift, splice site mutation, intragenic deletion) on both alleles of *SMARCB1* had inferior outcomes (57.8% ± [0.11] vs. 12% ± [0.07]). Furthermore the TYR-subgroup defined by 450 k methylation profiling appeared to confer a significant survival benefit (36.3% ± [0.15] vs. 25% ± [0.14]).

According to the presented data and ongoing research we suggest developing a risk-adapted strategic European approach for patients affected by AT/RT.

**References**

1. Atypical teratoid/rhabdoid tumors-current concepts, advances in biology, and potential future therapies. Frühwald MC, Biegel JA, Bourdeaut F, Roberts CW, Chi SN. Neuro Oncol. 2016;18(6):764–78.

2. Atypical Teratoid/Rhabdoid Tumors Are Comprised of Three Epigenetic Subgroups with Distinct Enhancer Landscapes. Johann PD, Erkek S, Zapatka M, Kerl K, Buchhalter I, Hovestadt V, Jones DT, Sturm D, Hermann C, Segura Wang M, Korshunov A, Rhyzova M, Gröbner S, Brabetz S, Chavez L, Bens S, Gröschel S, Kratochwil F, Wittmann A, Sieber L, Geörg C, Wolf S, Beck K, Oyen F, Capper D, van Sluis P, Volckmann R, Koster J, Versteeg R, von Deimling A, Milde T, Witt O, Kulozik AE, Ebinger M, Shalaby T, Grotzer M, Sumerauer D, Zamecnik J, Mora J, Jabado N, Taylor MD, Huang A, Aronica E, Bertoni A, Radlwimmer B, Pietsch T, Schüller U, Schneppenheim R, Northcott PA, Korbel JO, Siebert R, Frühwald MC, Lichter P, Eils R, Gajjar A, Hasselblatt M, Pfister SM, Kool M. Cancer Cell. 2016 14;29(3):379–93.

3. Improved 6-year overall survival in AT/RT - results of the registry study Rhabdoid 2007. Bartelheim K, Nemes K, Seeringer A, Kerl K, Buechner J, Boos J, Graf N, Dürken M, Gerss J, Hasselblatt M, Kortmann RD, Teichert von Luettichau I, Nagel I, Nygaard R, Oyen F, Quiroga E, Schlegel PG, Schmid I, Schneppenheim R, Siebert R, Solano-Paez P, Timmermann B, Warmuth-Metz M, Frühwald MC. Cancer Med. 2016 Aug;5(8):1765–75.

## A20 Recessively-acting choline transporter mutations associated with severe neurodevelopmental delay and congenital hypotonia

### Haicui Wang^1^, Holly Hardy^2^, Osama Refai^3^, Katy E. S. Barwick^2^, Holly H. Zimmerman^4^, Joachim Weis^5^, Emma L. Baple^2^, Andrew H Crosby^2^, Sebahattin Cirak^1^

#### ^1^Uniklinik Köln, Klinik für Kinderheilkunde und Jugendmedizin, Köln, Germany; ^2^RILD Wellcome Wolfson Centre, Royal Devon & Exeter NHS Foundation Trust, Barrack; Road, Exeter, UK; ^3^University of Calgary, Calgary, Canada; ^4^University of Mississippi, Medical Center of Jackson, Jackson, MS, USA; ^5^Uniklinik Aachen, Institut für Neuropathologie, Aachen, Germany

Haicui Wang and Holly Hardy are contributed equally to this study

Andrew H Crosby and Sebahattin Cirak are Co-Senior authors

##### **Correspondence:** Sebahattin Cirak

The high-affinity choline transporter (CHT, encoded by *SLC5A7*) is a critical determinant of acetylcholine signalling at the specialized neuromuscular junctions (NMJ) and central cholinergic synapses. Here we describe an autosomal recessive form of severe congenital hypotonia associated with CHT N-terminal missense mutations. The patients presented with severe neurodevelopmental delay and hypotonia indicating the dysfunction of the central synapses. Patient muscle biopsies revealed targetoid-like lesions. Whole exome sequencing revealed co-segregating *SLC5A7* mutations in two families with in total 3 patients. Each sequence mutation (NM_021815.2, c.282 T > A / p.Ser94Arg in Family 1, c.335 T > A, p.Val112Glu in Family 2) result in a single amino acid change in the transmembrane spanning domain. Each substitution affects residues which display high evolutionarily sequence conservation show high damage prediction, and alter residues located in the intracellular loop regions located between the third – sixth transmembrane domains. *In vivo* pan-neuronal expression of the wild type and mutant CHT in Caenorhabditis elegans revealed a complete loss of axonal transport associated with CHT mutation. Consistent with this our choline transport cell transfection assays and immunohistochemical studies demonstrated significant reductions in CHT cell surface levels, as well as a severe reduction in transporter activity associated with CHT mutation. These findings contrast with our previous description of a dominant-negative frameshift mutation at the C-terminus of CHT, associated with significantly reduced but not completely abrogated CHT transporter function leading to autosomal dominant distal hereditary motor neuropathy (dHMN). Together our findings define distinct neurological outcomes, disease processes, and modes of inheritance arising from different classes of CHT mutation and provide invaluable insight into the biological role of CHT as well as NMJ dysfunction.

## A21 Cord blood metabolome is associated to birth weight, but not predictive for weight gain and later BMI^A^

### Hellmuth C.^1^, Uhl O.^1^, Standl M.^2^, Heinrich J.^2, 3^, Thiering E.^2^, Koletzko B.^1^

#### ^1^Ludwig-Maximilian-Universität Munich, Div. Metabolic and Nutritional Medicine, Dr. von Hauner Children’s Hospital, University of Munich Medical Center, Munich, Germany; ^2^Institute of Epidemiology I, Helmholtz Zentrum München- German Research Center for Environmental Health, Neuherberg, Germany; ^3^Institute and Outpatient Clinic for Occupational, Social and Environmental Medicine, Inner City Clinic, University Hospital Munich, Ludwig Maximilian University of Munich, Munich, Germany

Obesity and its consequences are increasing challenges for developed and developing countries. The exposures to maternal factors like obesity or diabetes in-utero may affect offspring metabolism and already set the route for later obesity. As a consequence fetal metabolism may be changed. Cord blood metabolites reflect fetal growth and might also serve as predictors of infant growth patterns and childhood obesity risk. We aimed to characterize associations of metabolites assessed in >700 cord blood samples from infants participating in the German birth cohort study LISAplus with birth weight, postnatal weight gain, and BMI in adolescence. We used a targeted metabolomics platform for quantification of cord blood metabolites, including polar lipids, acylcarnitines, amino acids and non-esterified fatty acids (NEFA) and glycerophospholipid fatty acids (GPL-FA). Associations between metabolite concentrations in cord blood to birth weight, weight gain and BMI at 2 and 15 years of age were determined using linear regression models adjusted for confounding variables. In total, 581 metabolites were measured of which 209 passed the quality control procedures (CV < 30%). Cord blood metabolites were highly associated with birth weight. Lysophosphatidylcholines C16:1, C18:1, C20:3, C18:2, C20:4, C14:0, C16:0, C18:3, GPL-FA C20:3n-9, and GPL-FAC22:5n-6 were positively related to birth weight, while higher cord blood concentrations of NEFA C22:6, NEFA C20:5, GPL-FA C18:3n-3 and PCe C38:0 were associated with lower birth weight. In contrast, postnatal weight gain and BMI z-scores were not significantly associated with cord blood metabolites after adjustment for multiple testing. We conclude that cord blood metabolites are closely associated with birth weight and reflect intra-uterine development, but do not predict later weight gain and obesity risk at school age. A similar version of this abstract has been published before [A].

Funding sources: The LISAplus study was mainly supported by grants from the Federal Ministry for Education, Science, Research and Technology. The metabolomics analyses were supported by the European Research Council Advanced Grant META-GROWTH (ERC-2012-AdG – no.322605).

This abstract has been published [1] and is reproduced here with kind permission of S. Karger AG.

**Reference**

1. A. Ann Nutr Metab 2016;69:99–118 (DOI: 10.1159/000450569)

## A22 Targeting primary ciliogenesis in atypical teratoid/rhabdoid tumors

### Lena Blümel^1,2,3^, Kornelius Kerl^4^, Daniel Picard^1,2,3^, Michael C. Frühwald^5^, Max C. Liebau^6^, Guido Reifenberger^3^, Arndt Borkhardt^1^, Martin Hasselblatt^7^, Marc Remke^1,2,3^

#### ^1^Department of Pediatric Oncology, Hematology and Clinical Immunology, Heinrich Heine University Düsseldorf, Düsseldorf, Germany; ^2^Division of Pediatric Neuro-Oncogenomics, German Cancer Consortium and German Cancer Research Center - partner site Essen/Düsseldorf, Düsseldorf, Germany; ^3^Institute of Neuropathology, Heinrich Heine University Düsseldorf, Düsseldorf, Germany; ^4^Department of Pediatric Hematology and Oncology, University Hospital Münster, Münster, Germany; ^5^Swabian Childrens’ Cancer Center, Children’s Hospital Augsburg, Augsburg, Germany; ^6^Department of Pediatrics and Center for Molecular Medicine, University Hospital Cologne, Cologne, Germany; ^7^Institute of Neuropathology, University Hospital Münster, Münster, Germany

**Background**

Atypical teratoid/rhabdoid tumors (ATRT) are highly malignant CNS tumors commonly diagnosed in infants. Recently, comprehensive genomic studies defined three distinct molecular subtypes (TYR, MYC and SHH) of ATRT. Specifically, TYR-ATRT show overexpression of genes mediating ciliogenesis. As primary cilia have already been linked to the initiation and progression of other tumor entities, we aimed to characterize the distribution of primary cilia in ATRT, and to target primary ciliogenesis therapeutically in these tumors with dismal prognosis.

**Methods**

We analyzed primary cilia formation by immunofluorescence (IF) in several established ATRT cell lines and primary tumor sections (n = 13) using antibodies against pericentrin and acetylated tubulin staining the basal body and the axoneme of the primary cilium, respectively. The functional role of primary cilia in ATRT was examined by siRNA-mediated knockdown of ciliary proteins (KIF3A/IFT88) and by pharmacological inhibition using Ciliobrevin D, a cytoplasmic dynein inhibitor, known to target primary ciliogenesis.

**Results**

We detected primary cilia in all ATRT cell lines and primary tumor sections by IF. Notably, TYR-ATRT were highly ciliated (range 12-22%), while MYC-ATRT and SHH-ATRT displayed a variable degree (range 4-29%) and a low proportion (range 2-6%) of cells with primary cilia, respectively. Knockdown of ciliary proteins significantly reduced self-renewal capacity. Pharmacological inhibition of primary ciliogenesis phenocopied the siRNA-mediated knockdown results and the inhibitory effect was more pronounced after induction of primary ciliogenesis through serum starvation.

**Conclusion**

Primary cilia were previously regarded as rudimentary organelles, while a crucial role for primary cilia in cancer initiation and progression is now emerging. In all, our results implicate primary cilia as a universal feature of ATRT and suggest primary ciliogenesis as a potential therapeutic target especially in TYR-ATRT.

## A23 Inhibition of teneurin-2 (TENM2) leads to upregulation of UCP1 in human white adipocytes

### Tews D., Wabitsch M., Fischer-Posovszky P.

#### Division of Pediatric Endocrinology and Diabetes, Department of Pediatrics and Adolescent Medicine, University Medical Center Ulm, Ulm, Germany

**Background**

Heat generation in UCP1 active cells as present in brown adipose tissue contributes to the regulation of energy homeostasis in the context of weight regulation. Brown adipose tissue is known to be present in neonates and infants and has recently also been demonstrated in children and adults. Interestingly, a transition of white adipocytes into a brown phenotype has been documented *in vitro* in mouse and human cells, yet the underlying mechanisms are still not resolved. Using transcriptome analysis comparing human white and brown adipocyte progenitor cells, we identified Teneurin-2 (TENM2) as highly expressed in white progenitor cells.

In this study we tested whether TENM2 deficient preadipocytes convert into the brown/beige adipocyte lineage.

**Methods**

Human SGBS preadipocytes were transfected with siRNA against human TENM2 or control siRNA two days before inducing adipogenic differentiation. Markers of adipogenesis and brown adipocyte marker genes were analysed using qRT-PCR and Western Blot. Mitochondrial mass was quantified by measuring citrate synthase activity.

**Results**

During the course of adipogenesis, the mRNA expression on TENM2 was high in preadipocytes and decreased to nearly undetectable levels in adipocytes. Using siRNA we achieved a TENM2 knockdown by 70% in preadipocytes. Both TENM2 knockdown and control cells differentiated equally well into adipocytes as shown by quantification of adipogenic differentiation rates and adipocyte marker gene expression (PPARg, GLUT4, FASN). Their mitochondrial mass was comparable. The expression of the brown adipocyte marker gene UCP1 was significantly higher in TENM2 knockdown cells (mRNA 3-fold, protein 4-fold vs control).

**Conclusion**

Our data show that the downregulation of TENM2 leads to the induction of a brown adipocyte phenotype. We therefore conclude that TENM2 is a candidate gene for pharmacological interventions aiming at an induction of brown adipogenesis.

## A24 Optimizing multi-modular therapies for glioblastoma treatment

### Mike-Andrew Westhoff, Lisa Nonnenmacher, Julia Langhans, Lukas Schneele, Nancy Trenkler, Klaus-Michael Debatin

#### Department of Pediatrics and Adolescent Medicine, University Medical Center Ulm, Ulm, Germany

The RIST therapy is a multi-modular treatment consisting of rapamycin, irinotecan, sunitinib and temozolomide that has shown some considerable promise for difficult to treat malignancies in a compassionate use-setting. While investigating the individual components of this approach we found that replacing the mTOR-inhibitor rapamycin with the PI3K-blocker GDC-0941 greatly increased the therapeutic response of glioblastoma cells *in vitro*, but almost completely ablates the efficacy in an orthotopic mouse model. This is most likely due to additional effects of GDC-0941, i.e. inhibition of neovascularisation which leads to a reduction in delivery of chemotherapeutic agents to the tumour.

In-depth comparison between rapamycin and GDC-0941 revealed that, although different concentrations are needed for achieving target inhibition, both substances have a similar effect on proliferation and spontaneous apoptosis induction, while they synergize equally well with temozolomide. Importantly, used as single components in the animal model, only treatment with GDC-0941, but not with rapamycin, has a significant effect on overall survival.

In conclusion, we postulate that the more potent substance does not necessarily make a better component of a multi-modular therapy and multiple factors need to be evaluated prior to designing a new treatment approach.

